# Assessment of Inactivating Stop Codon Mutations in Forty *Saccharomyces cerevisiae* Strains: Implications for [*PSI* ^+^] Prion- Mediated Phenotypes

**DOI:** 10.1371/journal.pone.0028684

**Published:** 2011-12-15

**Authors:** David A. Fitzpatrick, Jennifer O'Brien, Ciara Moran, Naushaba Hasin, Elaine Kenny, Paul Cormican, Amy Gates, Derek W. Morris, Gary W. Jones

**Affiliations:** 1 Genome Evolution Laboratory, Department of Biology, National University of Ireland Maynooth, Maynooth, County Kildare, Ireland; 2 Yeast Genetics Laboratory, Department of Biology, National University of Ireland Maynooth, Maynooth, County Kildare, Ireland; 3 Trinity Genome Sequencing Laboratory, Institute of Molecular Medicine and Department of Psychiatry, Trinity College Dublin, Dublin, Ireland; University of Kent, United Kingdom

## Abstract

The yeast prion [*PSI* ^+^] has been implicated in the generation of novel phenotypes by a mechanism involving a reduction in translation fidelity causing readthrough of naturally occurring stop codons. Some [*PSI* ^+^] associated phenotypes may also be generated due to readthrough of inactivating stop codon mutations (ISCMs). Using next generation sequencing we have sequenced the genomes of two *Saccharomyces cerevisiae* strains that are commonly used for the study of the yeast [*PSI* ^+^] prion. We have identified approximately 26,000 and 6,500 single nucleotide polymorphisms (SNPs) in strains 74-D694 and G600 respectively, compared to reference strain S288C. In addition to SNPs that produce non-synonymous amino acid changes we have also identified a number of SNPs that cause potential ISCMs in these strains, one of which we show is associated with a [*PSI* ^+^]-dependent stress resistance phenotype in strain G600. We identified twenty-two potential ISCMs in strain 74-D694, present in genes involved in a variety of cellular processes including nitrogen metabolism, signal transduction and oxidative stress response. The presence of ISCMs in a subset of these genes provides possible explanations for previously identified [*PSI* ^+^]-associated phenotypes in this strain. A comparison of ISCMs in strains G600 and 74-D694 with *S. cerevisiae* strains sequenced as part of the *Saccharomyces* Genome Resequencing Project (SGRP) shows much variation in the generation of strain-specific ISCMs and suggests this process is possible under complex genetic control. Additionally we have identified a major difference in the abilities of strains G600 and 74-D694 to grow at elevated temperatures. However, this difference appears unrelated to novel SNPs identified in strain 74-D694 present in proteins involved in the heat shock response, but may be attributed to other SNP differences in genes previously identified as playing a role in high temperature growth.

## Introduction

The yeast non-mendelian genetic element [*PSI* ^+^] is a prion form of the translation termination protein Sup35p [1], [2]. The presence of [*PSI* ^+^] is routinely monitored by the increased readthrough of aberrant stop codons within the coding regions of useful genetic markers producing functional products [1]. Since the proposal that prions exist in yeast [2], a number of laboratories have contributed to prove this fact and have also identified molecular chaperones as the main cellular factors that influence prion propagation [3]–[6].

One area of major study is the evolutionary significance of prions in yeast. In addition to the relatively well-characterized yeast prions [*PSI* ^+^], [*URE3*] and [*PIN* ^+^]/[*RNQ* ^+^], recent evidence suggests the existence of a significant number of yeast proteins, involved in diverse cellular activities, that have the ability to form prions [7]–[12]. The existence of a large number of diverse proteins possessing the inherent ability to behave as prions suggests that there may be an evolutionary significance associated with prion-forming ability.

There is an ongoing debate in the yeast prion field as to the whether the [*PSI* ^+^] prion should be viewed solely as a protein mis-folding disease of yeast or that this prion may be of benefit to the cell under different environmental conditions [13]–[15]. The Sup35p prion forming domain, as well as those of other confirmed yeast prions, is conserved across diverse fungi [16]. A key question still under discussion is the nature of the selection pressure that maintains the presence of the Sup35p prion-forming domain throughout evolution. Whether this conservation is attributable to enhanced protein function or prion-forming ability remains to be resolved [17], [18]. Whatever the selection pressure for maintenance of the Sup35p prion-forming domain is, the presence of [*PSI* ^+^] in a yeast cell reduces translation termination fidelity and therefore has the potential to influence cellular phenotypes by causing readthrough of stop codons, be they naturally occurring stop codons or aberrant stop codons located within ORFs (so-called Inactivating Stop Codon Mutations- ISCMs). The potential of [*PSI* ^+^] to influence cellular phenotypes has been elegantly demonstrated in previous studies [15], [19]–[21]. To investigate the possible influence of ISCMs on [*PSI* ^+^] prion-dependent phenotypes at the molecular level we used next generation sequencing to obtain the genome sequence of two laboratory yeast strains (G600 & 74-D694) commonly used for the study of the yeast [*PSI* ^+^] and compared to the reference *S. cerevisiae* sequenced strain S288C. We identified SNPs between G600, 74-D694 and S288C and worked out the evolutionary relationship between these two newly sequenced strains and currently sequenced *S. cerevisiae* strains. The identification of a number of potential ISCMs in strains G600 and 74-D694 provides possible explanations for [*PSI* ^+^] prion dependent phenotypes in each strain. Comparison of ISCMs in previously sequenced yeast strains and the two [*PSI* ^+^] strains identifies huge variation in the generation of strain-specific ISCMs and highlights the potential for epigenetic regulation by aberrant stop codon readthrough. In addition, we provide insight into genotypic reasons why these two [*PSI* ^+^] harboring strains differ immensely in their ability to grow at elevated temperatures.

## Results

### Identification of SNPs in strains 74-D694 and G600

Following genomic DNA extraction and library construction we used next generation sequencing to characterize the genomes of [*PSI* ^+^] yeast strains 74-D694 and G600. Using an Illumina GAII we obtained approximately 40× genome coverage. Compared to the reference genome sequence of yeast strain S288C we identified totals of approximately 25,500 high quality SNPs in strain 74-D694 and 6,500 in G600. This equated to approximately 5,500 and 1,500 non-synonymous amino acid changes respectively (Table 1). A complete list of called SNPs for 74-D694 and G600 with corresponding amino acid changes can be found in Supplemental Tables S1 and S2 respectively.

**Table 1:** Summary of SNPs present in 74-D694 and G600 compared to S288C.

Strain	Size(bp)^a^	ORFs^a^	Total number of SNPs	SNPs in ORFs	Non- synonymous amino acid changes
74-D694	12,070,898	6,602	25,837	15,669	5,516
G600	12,070,898	6,602	6,551	4,077	1,549

a Information obtained from *Saccharomyces* Genome Database.

### Phylogenetic analysis of 74-D694 and G600

**Figure 1:**
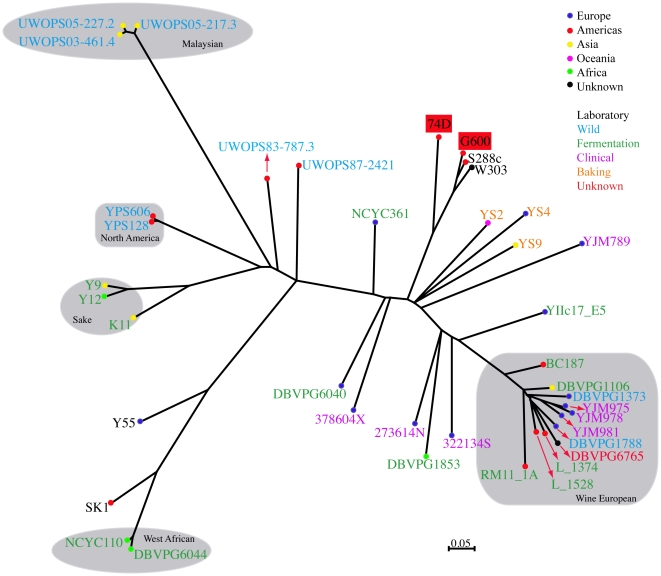
Phylogenetic relationship between 74-D694, G600 and *S. cerevisiae* strains sequenced as part of the SGRP. Phylogeny was constructed as described in Materials and Methods. Strain G600 is the most closely related sequenced yeast strain to the reference S288C strain.

To compare the relatedness of these two [*PSI* ^+^] strains to other sequenced *S. cerevisiae* strains we carried out phylogenetic analysis with strains sequenced as part of the *Saccharomcyes* Genome Resequencing Project [22]. Figure 1 shows that strain G600 is the most closely related currently sequenced yeast strain to the S288C reference strain. Strain 74-D694, although being more distantly related to S288C than G600, is still much less divergent than the vast majority of *S. cerevisiae* strains previously analyzed.

### Presence of ISCMs in diverse genes in 74-D694 and G600 explains some prion-associated phenotypes

In addition to non-synonymous amino acid changes we also identified twenty-two ISCMs in a wide variety of genes in strain 74D-694 (Table 2) and four in strain G600 (Table 3). Comparison of ISCMs in 74-D694 and G600 with ISCMs of other sequenced *S. cerevisiae* strains identified ISCMs that are specific to the two [*PSI* ^+^] strains. These strain-specific ISCMs are highlighted in Tables 2 and 3, and are most likely responsible for any [*PSI* ^+^] associated phenotypes in these strains. The SNP analysis correctly identified known nonsense mutations in strain 74-D694, namely *ade1-14* (W244TGA) and *trp1-289* (Q135TAG), and G600 *ade2-1* (E64TAA), and provided confidence that all other identified ISCMs would also be correct. However, to provide further evidence that all called ISCMs are correct, using PCR, we amplified 800–1000 bp of sequence flanking putative ISCMs in six genes (*DAL4, INP52, LAG1, MSN4, NSR1* and *SPH1*) and sequenced the PCR product using the Sanger method. All six ORFs were confirmed as containing the identified aberrant stop codons (data not shown). We took this as strong evidence for the efficacy of the genome sequencing and as confirmation of the presence of all ISCMs identified.

**Table 2:** ORFs in 74-D694 containing single internal nonsense mutations.

Systematic name^a^	Gene name^a^	Biological function^a^	Chromosomal SNP position	Nonsense change and consequence
YAL056W^b^	*GPB2*	Multistep regulator of c-AMP PKA signaling	41,780	Q841TAA39 amino acid truncation
YAR015W^b^	*ADE1*	Adenine biosynthesis	170,101	W244TGA62 amino acid truncation
YAR028W	*-*	Putative integral membrane protein, upregulated in response to DNA damage	185,308	W141TGA93 amino acid truncation
YAR031W	*PRM9*	Putative transmembrane protein	186,930	W34TAG264 amino acid truncation
YBL037W^b^	*APL3*	Alpha adaptin large subunit protein of the clathrin associated protein complex	150,039	L943TAA82 amino acid truncation
YDR007W^b^	*TRP1*	Tryptophan biosynthesis	462,241	Q135TAG89 amino acid truncation
YDR147W^b^	*EKI1*	Ethanolamine kinase	752,397	W257TAG277 amino acid truncation
YFR057W^b^	*-*	Uncharacterised- unknown function	269,422	Y125TAA26 amino acid truncation
YGR159C^b^	*NSR1*	Involved in pre-rRNA processing and ribosome biogenesis	806,553	E54TAA360 amino acid truncation
YGR249W	*MGA1*	Protein similar to heat shock transcription factor	989,281	Q410TAA46 amino acid truncation
YGR281W^b^	*YOR1*	Multidrug transporter	1,054,483	K552TAA925 amino acid truncation
YGR283W^b^	*-*	Predicted to be involved in ribosome biogenesis	1,059,136	L304TAG37 amino acid truncation
YHL003C^b^	*LAG1*	Involved in snythesis of ceramide	101,280	W200TGA211 amino acid truncation
YHR143W	*DSE2*	Involved in degradation of cell wall during cell division	385,718	L69TAA256 amino acid truncation
YIR028W^b^	*DAL4*	Allantoin permease- involved in nitrogen metabolism	409,511	W349TGA286 amino acid truncation
YKL062W^b^	*MSN4*	Transcriptional activator related to Msn2	323,577	Q236TAA394 amino acid truncation
YKR056W	*TRM2*	tRNA methyltransferase	549,185	W32TGA607 amino acid truncation
YLR313C	*SPH1*	Protein involved in shmoo formation	760,398	Q649TAG23 amino acid truncation
YNL065W	*AQR1*	Plasma membrane multidrug transporter	505,411	Q563TAG23 amino acid truncation
YOL060C^b^	*MAM3*	Protein required for normal mitochondrial morphology	214,535	K535TAA171 amino acid truncation
YOR183W	*FYV12*	Protein of unknown function	678,996	E42TAA87 amino acid truncation
YPL222W^b^	*FMP40*	Proposed to be involved in responding to stresses	130,893	Q245TAA443 amino acid truncation

a Information obtained from *Saccharomyces* Genome Database.

b ISCM unique to 74-D694.

**Table 3:** ORFs in G600 containing single internal nonsense mutations.

Systematic name^a^	Gene name^a^	Biological function^a^	Chromosomal SNP position	Nonsense change and consequence
YBR074W^b^	*-*	Putative metalloprotease	387,247	Q323TAA653 amino acid truncation
YNL106C^b^	*INP52*	Polyphosphatidylinositol phosphatase	422,546	W651TAG532 amino acid truncation
YOR128C	*ADE2*	Adenine biosynthesis	566,003	E64TAA507 amino acid truncation
YOR129C	*AFI1*	Arf3p polarisation-specific docking factor	566,901	E887TAA6 amino acid truncation

a Information obtained from *Saccharomyces* Genome Database.

b ISCM unique to G600.

The majority of the ISCMs identified in both strains have the potential to cause major truncations of proteins (Tables 2 and 3). In 74-D694 fourteen out of the twenty-two identified ISCMs would potentially truncate close to or in excess of 100 amino acids with eleven of these being close to or greater than 200 amino acid truncations. Hence, the presence of the identified ISCMs in 74-D694 has the potential to produce an effective null phenotype for a variety of genes.

Of the four identified ISCMs in G600 (Table 3), the E887TAA nonsense mutation in the *AFI1* gene would only produce a six amino acid truncation. Given this protein is 887 amino acids in length suggests that this ISCM may not have a major impact on protein function and may not impact severely on cell phenotypes. This proposal is supported by the fact that deletion of the whole *AFI1* gene does not have a major effect on cell fitness or produce any severe growth or stress phenotypes (*Saccharomyces* Genome Database). However, the remaining three ISCMs produce major truncations and would certainly render the truncated proteins produced non-functional or severely impaired (already confirmed for Ade2 protein).

The presence of ISCMs in a relatively large number and diverse set of genes in 74-D694 provides the opportunity for the presence of the [*PSI* ^+^] prion to potentially have a major impact on specific protein production and cellular phenotypes in this strain. Previous studies using 74-D694 have identified potential [*PSI* ^+^]-mediated phenotypes in this strain [15], [19], [20] and the identification of ISCMs in 74-D694 sheds light on such prion-mediated phenotypes (see Discussion).

### A stress-related phenotype in strain G600 is [*PSI* ^+^] prion-dependent

**Figure 2:**
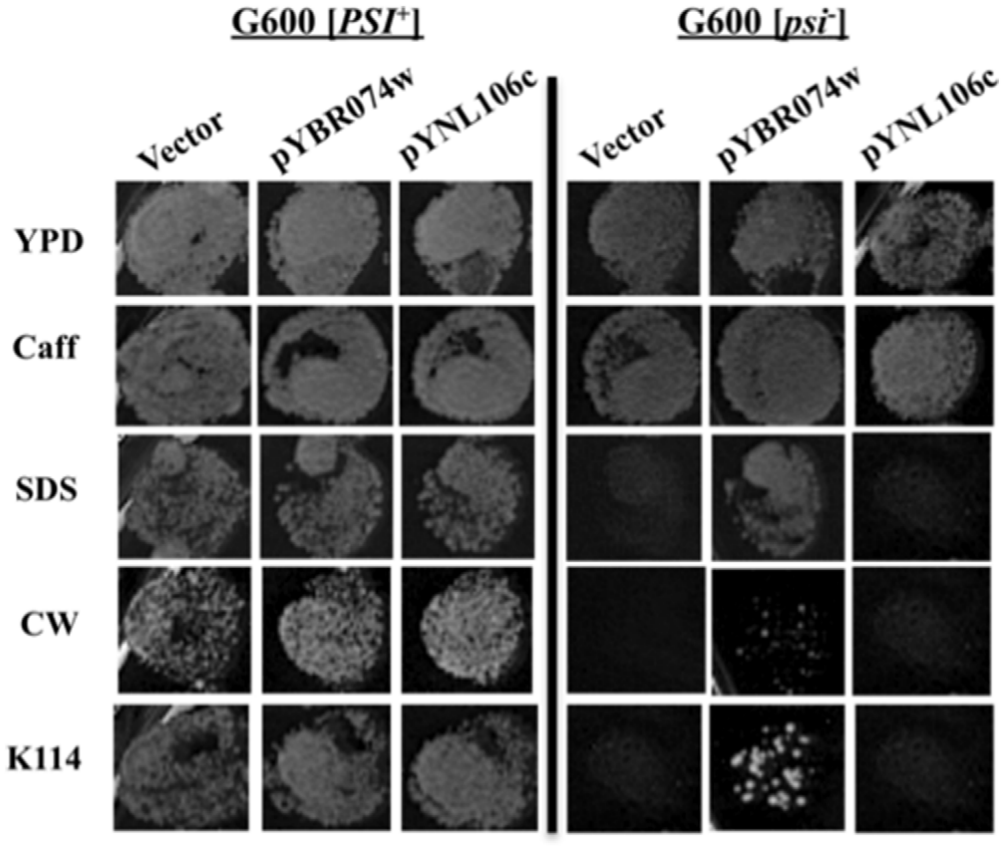
A stress phenotype in G600 is [*PSI* ^+^] dependent. G600 [*psi* ^−^] strains are more sensitive to some cell wall damaging agents than the [*PSI* ^+^] counterpart. The introduction of an ISCM-free copy of ORF YBR074W can partially complement [*PSI* ^+^] dependence in G600 for increased resistance to cell wall damaging agents (G600 [*psi* ^−^] middle panel). Caff = Caffeine (8 µM), SDS = Sodium Dodecyl Sulphate (0.005%), CW = Calcofluor White (10 µg/mL), K114 (Congo Red mimetic) = 10 µg/mL. Representative spots shown are neat concentrations from a serial dilution series.

Ongoing studies in our laboratory alluded to a possible [*PSI* ^+^]-dependent resistance phenotype to some cell wall damaging agents (C. Moran, unpublished observation). Given that the E64TAA nonsense mutation in *ADE2* and the potential relatively small six amino acids truncation in *AFI1* are both unlikely to be responsible for said phenotype, we concluded that either the potential truncations of YBR074W or YNL106C ORFs are the best possible candidates for causing this prion-dependent stress phenotype. To test this hypothesis we transformed [*PSI* ^+^] G600 with wild type copies of either YBR074W or YNL106C cloned into a plasmid vector. Following transformation we cured the strains and tested [*PSI* ^+^] and [*psi* ^−^] variants for resistance to cell wall damaging agents. Figure 2 shows that the presence of wild type YBR074W in [*psi* ^−^] G600 is able to confer increased resistance to SDS, the congo red mimetic chemical K114 and to a lesser extent calcofluor white. Thus the difference in resistance of [*psi* ^−^] and [*PSI* ^+^] variants of G600 to some cell wall damaging agents is in part due to the readthrough of the ISCM in the YBR074W gene present in this strain as reflected by the partial complementation of these phenotypes. Additionally a truncated version of YBR074W protein consisting of amino acid residues 1–322 only was unable to enhance growth of G600 [*psi* ^−^] on plates containing cell wall damaging agents (data not shown).

### Assessment of potential ISCMs in forty *S. cerevisiae* strains

The presence of twenty-two ISCMs in 74-D694 was initially surprising, as this number seemed rather large. We hypothesized that the fact that this strain harbored the [*PSI* ^+^] prion, the presence of which could increase stop codon readthrough, selection against the accumulation of ISCMs may be relaxed or compromised in strain 74-D694. To test this hypothesis we analyzed the presence of ISCMs present in *S. cerevisiae* strains sequenced as part of the SGRP. Using raw sequence data available from the Sanger Centre (http://www.sanger.ac.uk/research/projects/genomeinformatics/sgrp.html) we identified the total number of SNPs present in each of the thirty-eight sequenced strains (Supplementary Figure S1). We then identified the total number of ISCMs in each strain and compared this to each other SGRP strain and also to the [*PSI* ^+^] strains 74-D694 and G600 (Supplementary Table S5). Across the forty strains we identified 376 cases where a potential ISCM was present in an ORF. Some ISCMs are shared across the vast majority of the forty strains while others are confined to specific geographically defined groups of strains or to individual strains only (Supplementary Table S5).

**Figure 3:**
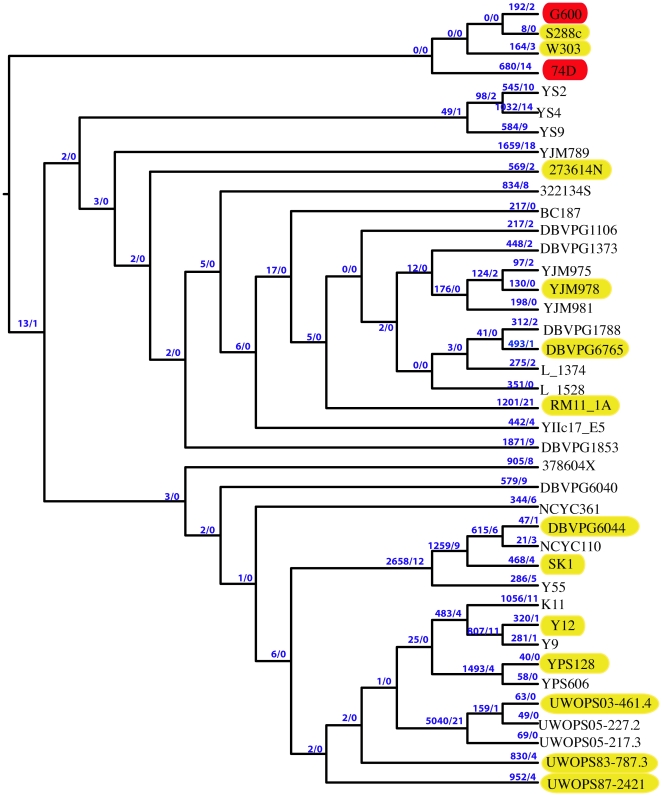
Strain and lineage unique non-synonymous SNPs and ISCMs in forty *S. cerevisiae* strains. Phylogenetic tree of forty sequenced *S. cerevisiae* strains highlighting the unique non-synonymous SNPs and ISCMs attributed to each strain and to each strain's lineage. Strains in red are [*PSI* ^+^], strains in yellow are confirmed [*psi* ^−^] strains. First number on a branch refers to the unique non-synonymous SNPs associated with a strain or branch. The second number represents the unique ISCMs associated with that strain or branch. For example, strain G600 contains 196 non-synonymous SNPs that are not found in any other of the 39 yeast strains, and of these 2 are unique ISCMs.

To assess the relative frequency of ISCMs in each of the forty strains we first identified the total number of strain specific SNPs. We then identified the total number of strain-specific non-synonymous and ISCM causing SNPs (Supplementary Figure S2). In addition, to assess the frequency and generation of ISCMs through *S. cerevisiae* evolution we also identified lineage specific SNPs. Figure 3 infers the phylogenetic relationship of the forty yeast strains analyzed in this study, lineage, branch and species specific ISCM and non-synonymous changes are also displayed. While the frequency of ISCMs per non-synonymous SNP is certainly high for strain 74-D694 (14/680 = 0.021) compared to the vast majority of the other *S. cerevisiae* strains, there are a number of strains that have comparable frequencies of ISCMs, even strains that are confirmed as being [*psi* ^−^]. Hence, the data suggests that many factors can influence the accumulation of ISCMs and currently we have no strong evidence to support the notion that the presence of [*PSI* ^+^] can cause an accumulation of ISCMs at an elevated rate as strains evolve. However, given that all the *S. cerevisiae* strains sequenced as part of the SGRP, bar the laboratory strains, were of diploid origin could reduce the biological significance of the ISCMs in these haploid segregants (see Discussion).

### Strains 74-D694 and G600 differ in ability to grow at elevated temperature but do have similar acquired thermotolerance capabilities

**Figure 4:**
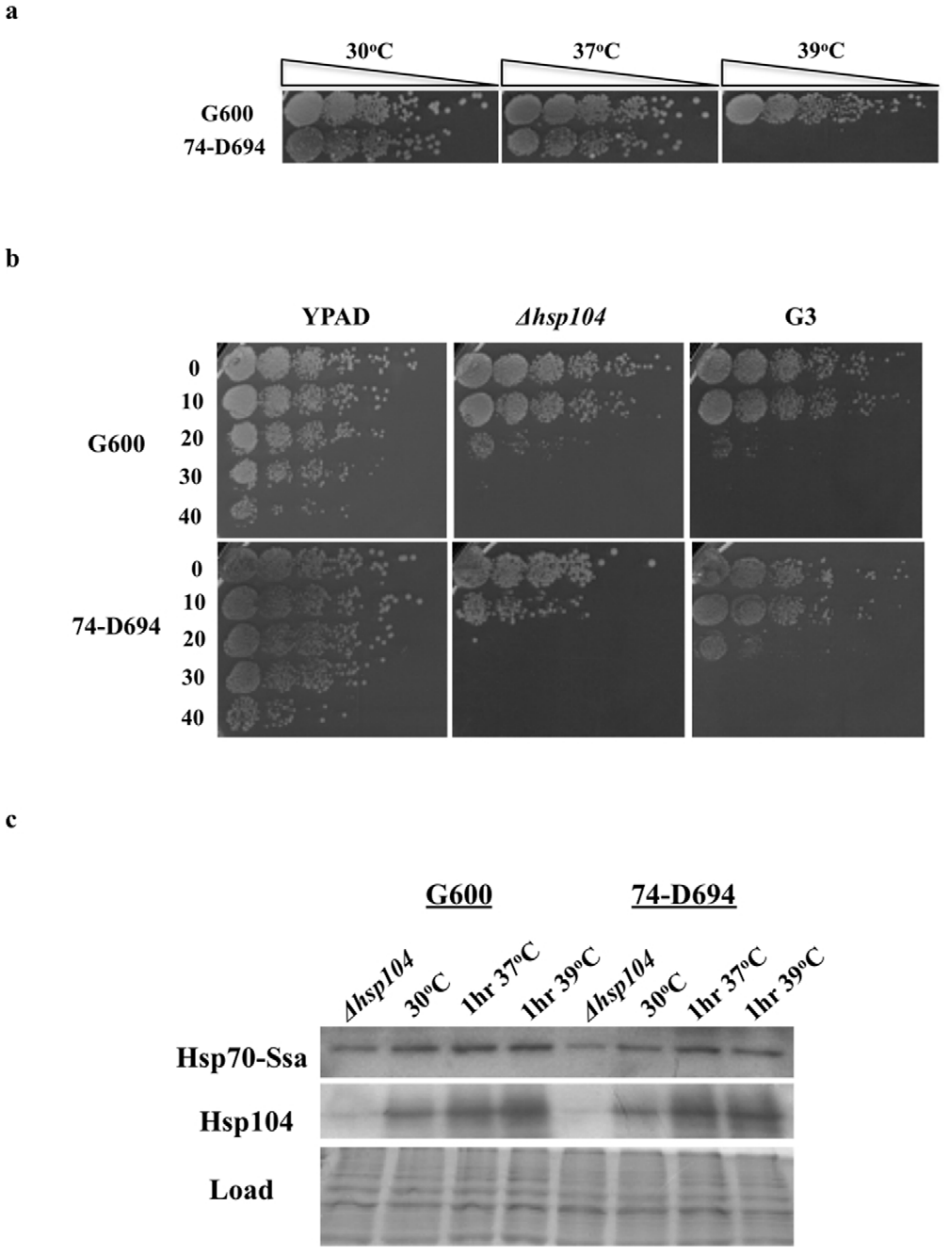
Ability to grow at elevated temperature differs between 74-D694 and G600, but heat-shock regulation seems similar. a) Spot dilution growth analysis demonstrates a clear difference in ability of 74-D694 and G600 to grow at 39°C. Growth is shown after 2-days incubation at the indicated temperatures. Following longer incubations 74-D694 is still unable to grow at 39°C. b) Acquired thermotolerance assay for 74-D694 and G600. Following induction of Hsp104p at 39°C for 1-hour, 74-D694 and G600 show similar recovery responses when exposed to 52°C for the indicated time periods. As shown previously the presence of 3 mM guanidine hydrochloride (G3) prevents this recovery by inhibiting Hsp104p. c) Immunoblot showing relative induction of Hsp70-Ssap and Hsp104p in strains 74-D694 and G600. Even though 74-D694 contains a number of non-synonymous amino acid differences in the heat shock master regulator Hsf1p, this does not appear to affect the ability to induce Hsp70-Ssa or Hsp104p to similar levels as G600.

It is well documented that molecular chaperones are key regulators of [*PSI* ^+^] propagation [3], [4], [6], and that single point mutations in chaperones can have major effects on prion propagation [23]–[26]. Therefore in both strains, we searched for amino acid changes in all chaperones and cochaperones that have been reported to influence prion propagation. Although we found no amino acid changes in the major prion influencing chaperone Hsp104p we did find non-synonymous amino acid changes in a number of chaperones and cochaperones known to influence [*PSI* ^+^] propagation (Supplemental Tables S3 and S4). However, apart from a shared polymorphism in the Hsp40 protein Apj1p (D524N), all chaperone polymorphisms are found in strain 74-D694, most notably twelve amino acid changes in the heat shock transcription factor Hsf1p (Supplemental Tables S3, S4 and S6). Given the presence of these polymorphisms in 74-D694 we compared the abilities of the two [*PSI* ^+^]- monitoring strains to grow at elevated temperatures. Figure 4a demonstrates that G600 has a much greater capacity than 74-D694 to grow at elevated temperatures, with the latter strain unable to grow at 39°C. We speculated that this difference may be due to altered regulation of the heat shock response in 74-D694 due to the presence of a large number of novel polymorphisms in Hsf1p. However, acquired thermotolerance is very similar in both strains (Figure 4b) as is the induction of Hsp104p in response to elevated temperature (Figure 4c). Hence, the inability of 74-D694 to grow at 39°C appears unrelated to regulation of the heat shock response in this strain.

In addition to polymorphic differences identified in heat shock response proteins between 74-D694 and G600, there is also a difference in a quantitative trait loci (QTL) associated with high temperature growth in yeast. Polymorphisms in genes *NCS2* and *MKT1* exhibit a complex genetic interaction that allows yeast strains to grow at high temperatures [27]. G600 contains the H71L polymorphism in *NCS2* that has been shown to be a major cause of heat resistance in yeast and also D30G and K453R polymorphisms in the *MKT1* gene (Supplemental Table S4). The *NCS2* allele is absent from 74-D694 (Supplemental Table S3) and may contribute to the extreme difference in the ability of these two strains to grow at elevated temperatures.

## Discussion

We have sequenced the genomes and identified SNPs in two strains of *S. cerevisiae* commonly used for studying the yeast [*PSI* ^+^] prion. To our knowledge these are currently the only two yeast strains sequenced that are confirmed as harboring [*PSI* ^+^]. Both strains are laboratory strains and this is reflected in their close evolutionary relationship to the *S. cerevisiae* reference strain S288C compared to other strains analyzed as part of the SGRP (Figure 1). Of the two [*PSI* ^+^] strains analyzed, G600 is by far more closely related to *S. cerevisiae* strains commonly used within the general yeast research community. In addition to the much greater number of non-synonymous amino acid changes in 74-D694 than G600 compared to the reference strain, 74-D694 also harbors a much greater number of ISCMs throughout its genome. This finding has major significance for this strain and its use for analysis of [*PSI* ^+^] related phenotypes. Potentially any internal stop codon mutation could affect protein production from the affected ORF. The presence of [*PSI* ^+^] in this strain allows for the possible readthrough of any internal stop codon mutation within an ORF and could therefore have implications for analysis of [*PSI* ^+^]- related phenotypes using this strain. Although harboring much fewer internal stop codon mutations, the same rationale needs to be applied to the G600 strain.

The identification of potential ISCMs in a variety of genes in strain 74-D694 may help to explain fully, or in part, some previously identified complex phenotypes associated with the presence or absence of [*PSI* ^+^] [15], [19], [20]. For instance, the presence of [*PSI* ^+^] has been shown to alter growth on nitrogen sources; we have identified an ISCM that could cause a major truncation in the Dal4p allantoin permease protein. The presence of [*PSI* ^+^] has been shown to alter cellular responses to exposure to metals, oxidative stress inducing agents and other stress inducing agents; we have identified ISCM in a number of genes that could be implicated in alteration of the cellular stress response. Most notably the presence of an ISCM in the *MSN4* gene could have major implications for strain 74-D694's ability to deal with oxidative stress. Msn4p is a transcription factor that works in conjunction with Msn2p to induce a variety of genes in response to oxidative cellular damage. However, there are a number of genes containing ISCMs in 74-D694 (Table 2) that are involved in (or potentially involved in) aspects of responding to DNA damage or stress, which could singularly or in combination contribute to complex cellular stress responses of 74-D694 in the presence or absence of [*PSI* ^+^]. Even though we currently have no data relating to the expected level of readthrough or alteration in mRNA stability of the ISCM containing genes, the identification of these potential ISCMs should be viewed as providing a framework for understanding or contributing significantly to elucidating the influence of [*PSI* ^+^] on prion-dependent phenotypes in this well-used laboratory strain.

Compared to reference strain S288C, G600 harbors far less potential ISCMs than 74-D694. In addition to the known E64TAA change in the *ADE2* gene [28] we identified ISCMs in ORFs YBR074W and YNL106C. The nonsense mutation in the *AFI1* gene would only cause a six amino acid truncation of the translated protein, with full-length protein being 887 amino acids and deletion of *AFI1* having a minimal effect on cell phenotype (*Saccharomyces* Genome Database) we predict this potential 6 amino acid truncation would not have much impact on the cell. A previously identified [*PSI* ^+^] prion-dependent phenotype has been shown to be at least partly attributable to readthrough of the ISCM in the uncharacterized YBR074W ORF (Figure 2). YBR074W is an uncharacterized yeast ORF that is predicted to be a metalloprotease [29]. Although we show a link between this gene and protection against chemicals that induce cell wall stress, we cannot provide any insight to the possible mechanistic role this protein may play in such protection. Thus, we now identify two [*PSI* ^+^] prion dependent phenotypes associated with readthrough of ISCMs in strain G600, adenine utilization and *ADE2* and cell wall stress response and YBR074W. In addition, given the common ancestry of G600 with other previously phenotypically characterized [*PSI* ^+^] strains (BSC783/4, 5V-H19 and 10B-H49) we can postulate that prion-dependent phenotypes identified in these strains may possibly result from readthrough of the two newly identified potential ISCMs in the G600 strain also being present in these strains [15].

We were initially surprised by the identification of what seemed a relatively large number of ISCMs in strain 74-D694. This raised the intriguing possibility that the presence of [*PSI* ^+^] in this strain had allowed the accumulation of ISCMs. Such a proposal is not unreasonable given Sup35p's role in translation termination and the reduction in fidelity in [*PSI* ^+^] strains. We therefore sought to assess the frequency of potential ISCMs in other sequenced *S. cerevisiae* strains. As part of the SGRP whole genome sequence data is available for thirty-eight *S. cerevisiae* strains. SNP analysis of these forty yeast strains shows a huge variation in the frequency of strain-specific ISCMs (Figure 3). Among the wild yeast a number of strains have accumulated zero strain-specific ISCMs whereas others have accumulated up to around 20. Taking into account the ISCM frequency per non-synonymous SNPs it appears that ISCM accumulation can be affected by an array of complex genetic and environmental factors. We could find no common genetic or environmental factors to account for the high (or low) occurrence of ISCMs in some wild yeast. From this analysis we could find no evidence to suggest that the presence of [*PSI* ^+^] could increase the rate of ISCM accumulation in evolving *S. cerevisiae*.

While our data suggest that the presence [*PSI* ^+^] does not influence the levels of ISCM accumulation, this proposal cannot be fully dismissed due to a number of considerations that need to be taken into account. Each non-laboratory strain sequenced as part of the SGRP is a haploid segregant from a diploid parent. Therefore the original parent strain has the potential to accumulate ISCMs without potentially altering protein function due to the extra copy of an altered gene. This fact could artificially increase the numbers of ISCMs in the wild yeast haploid segregants. Additionally the potential biological significance of an ISCM in these strains would only be observed if the original parent was homozygous for the ISCM. Such data is not available. The laboratory strains are all haploid and all have a relatively high frequency of ISCMs compared to the majority of the wild yeasts (Figure 3). The potential impact of [*PSI* ^+^] on ISCM accumulation could further be masked by growth in the laboratory environment [30]. Gu *et al* [30] have demonstrated that the rate of evolution appears higher in laboratory strains compared to wild yeast. This increased rate was attributed to a relaxed selection intensity, which could also impact on the accumulation of ISCMs. To fully address whether [*PSI* ^+^] can increase ISCM accumulation a controlled long-term evolution experiment with isogenic [*PSI* ^+^] and [*psi* ^−^] cells needs to be carried out.

Evidence has accrued to support the initial proposal by True and Lindquist [15] that [*PSI* ^+^] may allow evolutionary adaptation in specific growth conditions by allowing readthrough of naturally occurring stop codons [20], [31]–[34]. To assess whether we could find evidence for this at the genome level in the form altered C-terminal regions of proteins in [*PSI* ^+^] strains, we identified all altered naturally occurring stop codons in 74-D694, G600 and all the SGRP strains compared to S288C (data not shown). We found no evidence for alteration or extensions of C-terminal regions of proteins in the [*PSI* ^+^] strains. However, this is not surprising as any potential environmental adaptation caused by C-terminal extension of a protein would be transient and would not become fixed in the population unless the environmental selection persisted.

Using strain S288C as the reference strain means that by default we set the number of potential ISCMs in this strain as zero. However, this is not the case and S288C itself has some well- documented ISCMs (*Saccharomyces* Genome Database). We therefore assessed whether S288C ISCMs are shared in the two [*PSI* ^+^] strains and throughout the SGRP strains. Both 74-D694 and G600 harbor conserved ISCMs in the small ORF *CRS5* and in *FLO8* while ISCMs in ORFs *PAU7* and *PRP45* were present only in 74-D694. In addition an ISCM in *YNR066C* was present only in G600. However, absence or presence of S288C defined ISCMs in 74-D694 and G600 appears independent of the presence of [*PSI* ^+^] as they are also present in various SGRP strains (data not shown).

We noted that all ORFs containing potential ISCMs identified in strain G600 and 74-D694 are in non-essential genes. Theoretically it may be expected that the presence of [*PSI* ^+^] may allow the accumulation of ISCMs in essential genes also. However, given that [*psi* ^−^] derivatives of such mutations will most likely be inviable and this may result in the counter-selection against such mutations in the laboratory environment, which in turn may explain the apparent bias for ISCM generation in non-essential genes in these two strains.

Following the identification of twelve polymorphisms in the Hsf1 protein as well as a number of lesser changes in chaperone proteins known to influence prion propagation (all but one being absent from G600), we decided to assess the ability of G600 and 74-D694 to grow at elevated temperatures. Given the key role that chaperones play in modulating prion propagation, as attested to by research using both these [*PSI* ^+^] strains [23], [24], [26], [29], [35]–[38], any functional differences in chaperones due to the presence of identified polymorphisms will help to accurately assess the roles of these proteins in prion propagation in a strain- specific and more general manner. Although there is a major difference in the ability of G600 and 74-D694 to grow at 39°C (Figure 4a), there is no significant difference in the capability of these strains to acquire thermotolerance or to induce Hsp104 (Figures 4b and 4c). The ability of 74-D694 to grow at elevated temperatures is potentially further compromised by the absence of a QTL associated with high temperature growth. Previous studies have identified polymorphisms present in the *NCS2* and *MKT1* genes that can influence (in a background specific manner) growth at high temperature [27]. The associated QTLs are present in G600 and partly absent in 74-D694. Thus, although we identify chaperone polymorphism differences in G600 and 74-D694, these do not appear to be responsible for differences in these strains to grow at elevated temperatures. This finding suggests that overall the mechanism of heat shock response and prion propagation are similar in these two strains with respect to chaperone involvement.

Analyzing the genome of two [*PSI* ^+^] strains of *S. cerevisiae* has identified a number of ORFs harboring potential ISCMs. The presence of ISCMs in specific genes has allowed us to develop hypotheses for the causes of previously identified prion- associated phenotypes. In addition the genome and polymorphism data has allowed us to analyze the causes of differences in heat shock responses in these two strains. This genome data will allow researchers using these strains to develop and test hypotheses based on possible involvement of [*PSI* ^+^] (and perhaps other prions) in influencing phenotypic variation. Our study clearly shows that given the relative ease and low cost of obtaining yeast genome level data for specific strains, the information that can be amassed from such investment can have major impact on hypothesis driven research and experimental design.

## Materials and Methods

### Yeast strains, plasmids, media, genetic analysis and molecular methods

Yeast strains used in this study for genetical analysis are listed in Table 4. The G600 [*PSI* ^+^] strain is a descendent of the BSC strains originally characterized by Brian Cox. The 74-D694 [*PSI* ^+^] strain is a descendent of a Russian bakery strain unrelated to the sequenced S288C strain. The original parent strain was part of the Petershoff Genetic Lines collection and 74-D694 is derived from multiple crosses between the Petershoff strain and American laboratory strains related to S288C (Y. Chernoff, Personal Communication). Media is as previously described [26]. Plasmids pYCG_YBR074w and pYCG_YNL106c were purchased from Euroscarf and are cognate cloned plasmids for the designated yeast ORFs. These plasmids are based on *URA3* selection and thus pRS316 was used as the appropriate vector control. A plasmid harboring a truncated YBR074W gene was constructed as follows. Primers YBR074w-pC210F (AGATTTTATACAGAAATATTTATACATatgaaattaaaaagtgtattcagatc) and YBR074w-pC210R (TTCCCTGATTAAACAGGAAGACAAAGCATGCttataaggaagtatgtagcatatgcc) were used to PCR amplify the DNA sequence corresponding to amino acids 1–322 of the YBR074W protein. Regions of ORF homology within the primers are shown in lower case. Truncated YBR074W was cloned into *Nde*I and *Sph*I digested pC210 plasmid by gap-repair, regions of vector homology within the primers are shown in upper case. Gap repair was used due to the presence of an *Nde*I site within the truncated DNA sequence of the YBR074W ORF. Cloning into pC210 allows efficient expression of cloned ORFs from the constitutive *SSA2* promoter [39]. Sanger sequencing was used to confirm integrity of this construct. Spot growth assays were carried out by diluting an overnight culture of cells in fresh medium lacking uracil (-URA) to 1×10^6^ cells/mL. Cells were then grown to exponential phase to a density of 3×10^6^/mL. Cells were then re-suspended in -URA to a density of 5×10^6^/mL and transferred to a microtiter plate. Following a series of 1 in 5 dilutions cells were transferred to -URA plates using a multi- pronged replicator and incubated at the appropriate temperature for 2 days. For thermotolerance assays, exponentially growing cells were exposed to 39°C for 1-hour to induce Hsp104p. Cells were then exposed to 52°C for indicated time periods prior to transfer to microtiter plate and dilution analysis. Immunoblotting and antibodies for Hsp70-Ssap and Hsp104p was as previously described [26].

**Table 4:** Strains used in this study.

Strain name	Genotype	Source
G600	*MAT* **a** *ade2.1 SUQ5 kar1-1 his3 leu2 trp1 ura3* [*PSI^+^*]	[43]
74-D694	*MAT* **a**, *ade1-14, trp1-289, his3Δ200, ura3-52, leu2, 113* [*PSI* ^+^]	[35]
UCC8344	*MAT* ***α*** derivative of RM11	[44]
NCYC3609	*MAT* ***α*** derivative of UWOPS87-2421	[45]
NCYC3611	*MAT* ***α*** derivative of 273614N	[45]
NCYC3614	*MAT* ***α*** derivative of UWOPS83-787.3	[45]
NCYC3615	*MAT* ***α*** derivative of SK1	[45]
NCYC3617	*MAT* ***α*** derivative of YJM978	[45]
NCYC3622	*MAT* ***α*** derivative of DBVPG6765	[45]
NCYC3625	*MAT* ***α*** derivative of DBVPG6044	[45]
NCYC3627	*MAT* ***α*** derivative of UWOPS03-461.4	[45]
NCYC3630	*MAT* ***α*** derivative of Y12	[45]
NCYC3632	*MAT* ***α*** derivative of YPS128	[45]

#### Illumina library construction and sequencing

Genomic DNA was isolated from strains 74-D694 and G600 using a standard protocol. Libraries were generated using 1.5 µg of genomic DNA following the Illumina Genomic DNA sample preparation protocol with the exception that the samples were sheared using a biorupter (Diagenode) instead of nebulisation. (The samples were sonicated on high for 30 seconds and off for 30 seconds for a total 30 minutes with addition of ice after every 10 minutes to keep the samples cool). 16 pM of the sequencing library was loaded onto the flowcell and single reads of 40 bases were generated using an Illumina GAII.

The images were processed into sequence data and aligned to the *S. cerevisiae* genome using the Illumina GA Pipeline v1.4. SNP detection and generation of data to determine sequence coverage across the genome was performed with Mapping and Assembly with Quality (MAQ) using the default settings [40]. The Illumina quality scores were converted to the standard phred scores required by MAQ by using a modified version of the fq_all2std.pl script supplied with MAQ. Raw sequence data for 74-D694 can be retrieved from the following web address, http://bioinf.nuim.ie/wp-content/uploads/2011/10/74D_sequence.txt.zip and data for G600 from http://bioinf.nuim.ie/wp-content/uploads/2011/10/G600_sequence.txt.zip.

### Bioinformatics analysis of SNP data

Genome assemblies and associated SNP information for 38 *S. cerevisiae* strains were downloaded from the *Saccharomyces* Genome Resequencing Project (SGRP) FTP website (ftp://ftp.sanger.ac.uk/pub/dmc/yeast/latest). All SNPs have been called relative to the reference *S. cerevisiae* strain (S288C) [22]. Taking each re-sequenced strain in isolation, we determined whether SNPs occurred in coding or non-coding regions. If a SNP occurred in a coding region we subsequently determined whether it resulted in a synonymous or non-synonymous substitution. We also determined whether SNPs, non-synonymous substitutions and ISCMs were lineage or species specific. These were represented on our phylogenetic tree (Figure 3 and Supplemental Information). This process was repeated for all 38 SGRP strains and our two newly re-sequenced strains (74-D694 & G600).

### Confirmation of nonsense mutations in selected ORFs

Using PCR we amplified approximately 800–1000 bp regions flanking six putative ISCMs in different ORFs (*DAL4, INP52, LAG1, MSN4, NSR1* and *SPH1*). These fragments were sequenced using the Sanger method (LGC Genomics). All Sanger sequencing data for these six ORFs matched exactly to sequencing data retrieved using Illumina sequencing.

### Phylogenetic analysis

A maximum likelihood phylogenetic tree based on SNP differences was reconstructed for the forty *S. cerevisiae* strains used in this analysis. The appropriate nucleotide substitution model was selected by the ModelGenerator software [41]. One hundred bootstrap replicates were performed in Phyml [42] and summarized using the majority-rule consensus method.

## Supporting Information

Figure S1 Summary of total number and chromosome distribution of SNPs identified in 74-D694, G600 and thirty-eight SGRP *S.cerevisiae* strains.
(XLS)

Figure S2 Details of unique synonymous and non-synonymous SNPs allocated to each branch of the *S. cerevisiae* phylogenetic tree. Unlike figure 3 the number of ISCMs represented are absolute numbers for each strain or branch and is regardless of whether multiple nonsense mutations occur in a single ORF or whether the same ORFs in different strains contain different ISCMs. The two confirmed [*PSI* ^+^] strains are highlighted in red while confirmed [*psi* ^−^] strains are in yellow. An example of reading this table and diagram is as follows: branch number 46 shows there are 348 unique SNPs shared between strains YJM975 and YJM978 that are not found in any other of the 38 *S. cerevisiae* strains. Of these total SNPs 124 produce non-synonymous changes. Of these non-synonymous changes 2 produce potential ISCMs.
(XLS)

Table S1 Detailed breakdown of SNPs and non-synonymous amino acid changes by chromosomal location for strain 74-D694.
(DOC)

Table S2 Detailed breakdown of SNPs and non-synonymous amino acid changes by chromosomal location for strain G600.
(DOC)

Table S3 Complete list of all high quality SNPs and non-synonymous amino acid changes identified in strain 74-D694.
(TXT)

Table S4 Complete list of all high quality SNPs and non-synonymous amino acid changes identified in strain G600.
(TXT)

Table S5 **Heatmap-ISCMs**- heatmap detailing potential ISCMs identified in 74-D694, G600 and thirty-eight SGRP *S.cerevisiae* strains. The presence of a potential ISCM in a particular gene is represented by 1 and absence by 0.
(XLS)

Table S6 Summary of non-synonymous amino acid changes identified in chaperone proteins in strain 74-D694.
(DOC)
