# Supplementary material for: Assessment of Inactivating Stop Codon Mutations in Forty Saccharomyces cerevisiae Strains: Implications for [PSI +] Prion- Mediated Phenotypes
Source: PLoS One. 2011 Dec 15;6(12):e28684. doi: 10.1371/journal.pone.0028684 (PMC3240633; doi:10.1371/journal.pone.0028684)
Supplement: Table S1 — Detailed breakdown of SNPs and non-synonymous amino acid changes by chromosomal location for strain 74-D694. (DOC) [file pone.0028684.s003.doc]

**Table S1** – SNPs present in 74-D694 compared to reference strain S288C

| Chromosome | Size  (bp)^a^ | ORFs^a^ | Total number of SNPs | SNPs in ORFs | Non- synonymous amino acid changes |
| --- | --- | --- | --- | --- | --- |
| I | 230,208 | 117 | 923 | 559 | 200 |
| II | 813,178 | 456 | 1443 | 955 | 309 |
| III | 316,616 | 183 | 614 | 306 | 122 |
| IV | 1,531,919 | 836 | 2243 | 1380 | 482 |
| V | 576,869 | 324 | 1156 | 590 | 193 |
| VI | 270,148 | 141 | 507 | 245 | 86 |
| VII | 1,090,947 | 583 | 2611 | 1588 | 627 |
| VIII | 562,643 | 321 | 1678 | 1080 | 386 |
| IX | 439,885 | 241 | 1907 | 1065 | 383 |
| X | 745,741 | 398 | 1284 | 808 | 310 |
| XI | 666,454 | 348 | 1925 | 1117 | 365 |
| XII | 1,078,175 | 578 | 1752 | 1118 | 342 |
| XIII | 924,429 | 505 | 1533 | 978 | 368 |
| XIV | 784,333 | 435 | 1846 | 1147 | 411 |
| XV | 1,091,289 | 598 | 2619 | 1659 | 566 |
| XVI | 948,062 | 510 | 1584 | 1007 | 361 |
| mito | 85,779 | 28 | 212 | 67 | 5 |
| Totals | 12,070,898 | 6,602 | 25,837 | 15,669 | 5,516 |

^a^Information obtained from *Saccharomyces* Genome Database
