# Supplementary material for: Assessment of Inactivating Stop Codon Mutations in Forty Saccharomyces cerevisiae Strains: Implications for [PSI +] Prion- Mediated Phenotypes
Source: PLoS One. 2011 Dec 15;6(12):e28684. doi: 10.1371/journal.pone.0028684 (PMC3240633; doi:10.1371/journal.pone.0028684)
Supplement: Table S2 — Detailed breakdown of SNPs and non-synonymous amino acid changes by chromosomal location for strain G600. (DOC) [file pone.0028684.s004.doc]

**Table S2 – SNPs present in G600 compared to reference strain S288C**

| Chromosome | Size  (bp)^a^ | ORFs^a^ | Total number of SNPs | SNPs in ORFs | Non- synonymous amino acid changes |
| --- | --- | --- | --- | --- | --- |
| I | 230,208 | 117 | 157 | 126 | 59 |
| II | 813,178 | 456 | 885 | 516 | 211 |
| III | 316,616 | 183 | 228 | 157 | 65 |
| IV | 1,531,919 | 836 | 38 | 28 | 22 |
| V | 576,869 | 324 | 47 | 34 | 23 |
| VI | 270,148 | 141 | 32 | 14 | 7 |
| VII | 1,090,947 | 583 | 625 | 340 | 120 |
| VIII | 562,643 | 321 | 10 | 5 | 2 |
| IX | 439,885 | 241 | 505 | 304 | 98 |
| X | 745,741 | 398 | 380 | 270 | 94 |
| XI | 666,454 | 348 | 515 | 312 | 117 |
| XII | 1,078,175 | 578 | 652 | 461 | 150 |
| XIII | 924,429 | 505 | 655 | 380 | 138 |
| XIV | 784,333 | 435 | 673 | 396 | 177 |
| XV | 1,091,289 | 598 | 983 | 618 | 228 |
| XVI | 948,062 | 511 | 79 | 54 | 34 |
| mito | 85,779 | 19 | 87 | 62 | 4 |
| Totals | 12,070,898 | 6,602 | 6,551 | 4,077 | 1,549 |

^a^Information obtained from *Saccharomyces* Genome Database
