# Supplementary material for: Assessment of Inactivating Stop Codon Mutations in Forty Saccharomyces cerevisiae Strains: Implications for [PSI +] Prion- Mediated Phenotypes
Source: PLoS One. 2011 Dec 15;6(12):e28684. doi: 10.1371/journal.pone.0028684 (PMC3240633; doi:10.1371/journal.pone.0028684)
Supplement: Table S6 — Summary of non-synonymous amino acid changes identified in chaperone proteins in strain 74-D694. (DOC) [file pone.0028684.s008.doc]

**Supplementary table 6- Chaperone and cochaperones implicated in prion propagation that contain mis-sense mutations in strain 74-D694 compared to reference strain S288C**

| Systematic name^a^ | Gene name^a^ | Biological function^a^ | Chromosomal SNP position(s) | Non- synonymous change(s) |
| --- | --- | --- | --- | --- |
| YAL005C | *SSA1* | ATPase involved in protein folding and nuclear localization signal (NLS)-directed nuclear transport; member of heat shock protein 70 (Hsp70) family; forms a chaperone complex with Ydj1; localized to the nucleus, cytoplasm, and cell wall | 141,186 | A83G |
| YGL073W | *HSF1* | Trimeric heat shock transcription factor, activates multiple genes in response to stresses that include hyperthermia; recognizes variable heat shock elements (HSEs) consisting of inverted NGAAN repeats; posttranslationally regulated | 369,170  369,356  369,366  369,603  369,669  369,884  370,320  370,350  370,644  370,650  370,740  371,247 | D139N  F201L  T204M  N283S  S305I  Q377K  S522F  P532L  W630L  N632S  S662W  A831V |
| YJR032W | *CPR7* | Peptidyl-prolyl cis-trans isomerase (cyclophilin), catalyzes the cis-trans isomerization of peptide bonds N-terminal to proline residues; binds to Hsp82 and contributes to chaperone activity | 491,334  491,782 | S87L  L237F |
| YNL064C | *YDJ1* | Protein chaperone involved in regulation of the Hsp90 and Hsp70 functions; involved in protein translocation across membranes; member of the DnaJ family | 507,059  506,921  506,907 | P14S  P60S  D64E |
| YNL077W | *APJ1* | Putative chaperone of the HSP40 (DNAJ) family; overexpression interferes with propagation of the [*PSI*^+^] prion | 482,040  482,961 | K217E  D524N |
| YOR027W | *STI1* | Hsp90 cochaperone, interacts with the Ssa group of the cytosolic Hsp70 chaperones; activates the ATPase activity of Ssa1; homolog of mammalian Hop protein | 381,777 | K242R |
| YPL106C | *SSE1* | ATPase that is a component of the heat shock protein Hsp90 chaperone complex; binds unfolded proteins; member of the heat shock protein 70 (Hsp70) family; localized to the cytoplasm | 350,651 | G541A |
| YPL240C | *HSP82* | Hsp90 chaperone required for pheromone signaling and negative regulation of Hsf1; docks with Tom70 for mitochondrial preprotein delivery; promotes telomerase DNA binding and nucleotide addition | 97,732 | N298K |

^a^Information obtained from *Saccharomyces* Genome Database
